# Dam characteristics associated with pre-weaning diarrhea in mink (*Neovison vison*)

**DOI:** 10.1186/s13028-018-0427-z

**Published:** 2018-11-12

**Authors:** Julie Melsted Birch, Jens Frederik Agger, Bent Aalbæk, Tina Struve, Anne Sofie Hammer, Henrik Elvang Jensen

**Affiliations:** 1 Department of Veterinary and Animal Sciences, Faculty of Health and Medical Sciences, University of Copenhagen, Ridebanevej 3, 1870 Frederiksberg C, Denmark; 2 Kopenhagen Diagnostics, Kopenhagen Fur, Langagervej 60, 2600 Glostrup, Denmark

**Keywords:** Epidemiology, Female impact, Greasy kits, Mastitis, Mink, Nursing period, Pre-weaning diarrhea, Risk factors, Sticky kits, Wet kit syndrome

## Abstract

**Background:**

Pre-weaning diarrhea (PWD) in mink, also known as “sticky kits”, is a frequently occurring syndrome in suckling mink kits on commercial mink farms. Outbreaks of PWD result in weakened kits, increased mortality and reduced growth and welfare as well as considerable economic losses for the farmers. The syndrome is regarded as multifactorial with a complex etiology, and studies have focused on associations with environment, management and dam characteristics. The present study was conducted from May to June 2015 and included 70 dams with mink litters with and without PWD. The aims were to examine associations between PWD and mastitis (bacterial infection and histological signs of inflammation or other lesions in the mammary gland), and to examine associations between PWD and other dam-related characteristics (age, litter size, body mass index, and weight and number of active mammary glands of the dam).

**Results:**

Using multivariable mixed logistic regression analyses with farm id as a random intercept, we found that the odds for PWD in the litter were significantly higher in 1 year old dams versus > 1 year old (OR = 13.3, CI 2.0–90.2, P = 0.01), higher if litter size observed after birth was > 5 kits versus ≤ 5 kits (OR = 16.5, CI 2.2–123.7, P = 0.01), higher if the number of active mammary glands per kit was ≤ 1.5 versus > 1.5 glands per kit (OR = 6.5, CI 1.2–36.0), P = 0.03), and higher in farms with high prevalence of PWD versus low prevalence (OR = 16.8, CI 2.9–97.6, P = 0.002). There were no significant associations between PWD and bacterial infection, histological signs of inflammation or other lesions of the mammary gland, body mass index or weight of mammary gland per kit.

**Conclusion:**

Pre-weaning diarrhea had a statistically significant association with age of the dam, litter size and the number of active mammary glands per kit. However, PWD was not associated with mastitis, body mass index and weight of mammary gland tissue per kit.

## Background

Pre-weaning diarrhea (PWD) in mink kits is a well recognized disease in farmed mink (*Neovison vison*) and appears as a syndrome commonly referred to as “sticky kits”, “wet kits” or “greasy kits” [1]. Every year, outbreaks occur on mink farms with considerable economic losses for the farmers as well as decreased welfare for the affected animals. The syndrome typically affects all kits in a litter and is characterized by a yellow-white diarrhea. Concurrently, a greasy exudate on the skin spreads from the neck over the back and trunk and out on the limbs, claws and tail. The anal region becomes red and swollen, and if the diarrhea is severe, dehydration develops [1, 2]. The number of affected farms and the severity within the farms varies across years. The morbidity may reach over 30% of litters on a farm [1]. Affected mink kits show distressed behavior. Average mortality may vary between less than 1 and up to 2 kits per litter [1]. Studies have been focusing on the factors, which are associated with PWD. Several viruses have been identified in diarrheic mink kits including astrovirus, calicivirus, coronavirus and rotavirus [3–9], but most of these viruses have also been found in healthy mink kits [3, 4, 7].

Studies of bacteria from intestines of mink kits with PWD, have mainly focused on *Escherichia coli* and *Staphylococcus* spp. [7–10]. Both hemolytic and non-hemolytic *E. coli* strains have been isolated from diarrheic and healthy kits. These were further characterized, but differences in serotypes or virulence factors between diseased and non-diseased kits have not been identified [7, 10]. Therefore, the strains of *E. coli* are considered as opportunistic pathogens in weakened kits [7]. PWD has been associated with *Staphylococcus delphini* [9] and *Staphylococcus intermedius* [8]. The *S. intermedius* group (SIG) was reclassified and holds *S. delphini* group A [11]. However, mink are natural hosts of *S. delphini* and healthy mink kits host hemolytic staphylococci in their guts especially at the beginning of the nursing period [12, 13]. Hence, the microbial pattern associated with PWD is somewhat ambiguous. In general, the syndrome should be regarded as a multifactorial disease complex [1, 2], where factors such as feed producer, climate and population density have been reported to be significantly associated with the condition [2]. Dam-associated factors are also of concern for the development of PWD. Severe weight loss of the dam and restriction of feed allowance during the winter and late gestation period have been shown to increase the risk of PWD [14–16]. Large litters and litters from young dams have been found to have increased risk of PWD [15], and the immune status of the dam may also be of importance for development of PWD [17, 18]. Concurrent with PWD, mastitis is often a problem on the farms and has been hypothesized to be associated with the syndrome [2]. Moreover, mastitis has been reported to be associated with increased mortality in dams as well as in mink kits during the lactation period [19, 20]. Clinical manifestations of mastitis in mink dams are decreased appetite, lethargy and swelling of mammary gland(s). Bacterial cultures from mammary tissue often reveal *E. coli* or *Staphylococcus aureus* [20, 21], however, some of the staphylococci reported as *S. aureus* by these authors may have included other *Staphylococcus* species, which were later described. The pathology of *Staphylococcus*-associated mastitis typically shows a suppurative inflammation with abscessation, whereas mastitis caused by *E. coli* tends to be fibrino-necrotizing [21]. However, classical signs of inflammation are rarely seen in mink with mastitis and no method has been developed to diagnose subclinical mastitis in mink [19, 22]. Furthermore, it has been suggested that the prevalence of subclinical mastitis and its impact on mink health have been underestimated on mink farms [19, 21]. A study on an experimental farm enclosing 12 females with and without pre-weaning diarrhea did not show an association between PWD and mastitis [22], but no studies have been done on a greater spectrum of farms and have not evaluated if mastitis could be a risk factor among several others, which have been identified in this disease complex. The aims of this study were to examine associations between PWD in the mink litter and the presence of mastitis in terms of bacterial infection and histological signs of inflammation in the mammary gland of the dam, and to examine associations between PWD in the litter and other characteristics concerning the dam: age, litter size, body mass index (BMI), weight and number of mammary glands per kit, and the presence of other pathologic manifestations.

## Methods

### Study design and animals

The study was conducted in the nursing period from May 11 to June 2, 2015. Seventy mink dams and their litter of mink kits used for this study were sampled in a two stage sampling process from 25 Danish Aleutian mink disease (AMDV) free farms. In stage one, 10 farms were conveniently selected among farms with a high prevalence of PWD and 15 farms were conveniently selected among farms with a low prevalence of PWD. In stage two, dams and mink kits from litters with clinical signs of PWD and dams and their litters without clinical signs of PWD were conveniently sampled by the investigation team within the farms among litters presented by the farmer. The dam was then sedated with 12.5 mg ketamine and 5 mg xylazine given intramuscularly in a hindleg and euthanized by an intrahepatic injection of pentobarbital. From each litter, 2–3 kits were haphazardly selected and euthanized with an overdose of pentobarbital injected into the liver. Afterwards, the necropsies were carried out. The final categorization of diseased (PWD+) and non-diseased litters (PWD−) was based on the clinical assessment combined with the post mortem examination of the intestinal contents in the rectum, or at post mortem “defecation”. This was done in order to assure that mink litters with kits having liquid feces but no external signs of diarrhea were allocated to the PWD+ group. None of the dams or kits had been treated with antibiotics. Hence, the PWD+ group consisted of 29 litters (n = 29) and, the PWD− group consisted of 41 litters (n = 41). The study unit was the litter.

### Assessment of PWD status

**Fig. 1:**
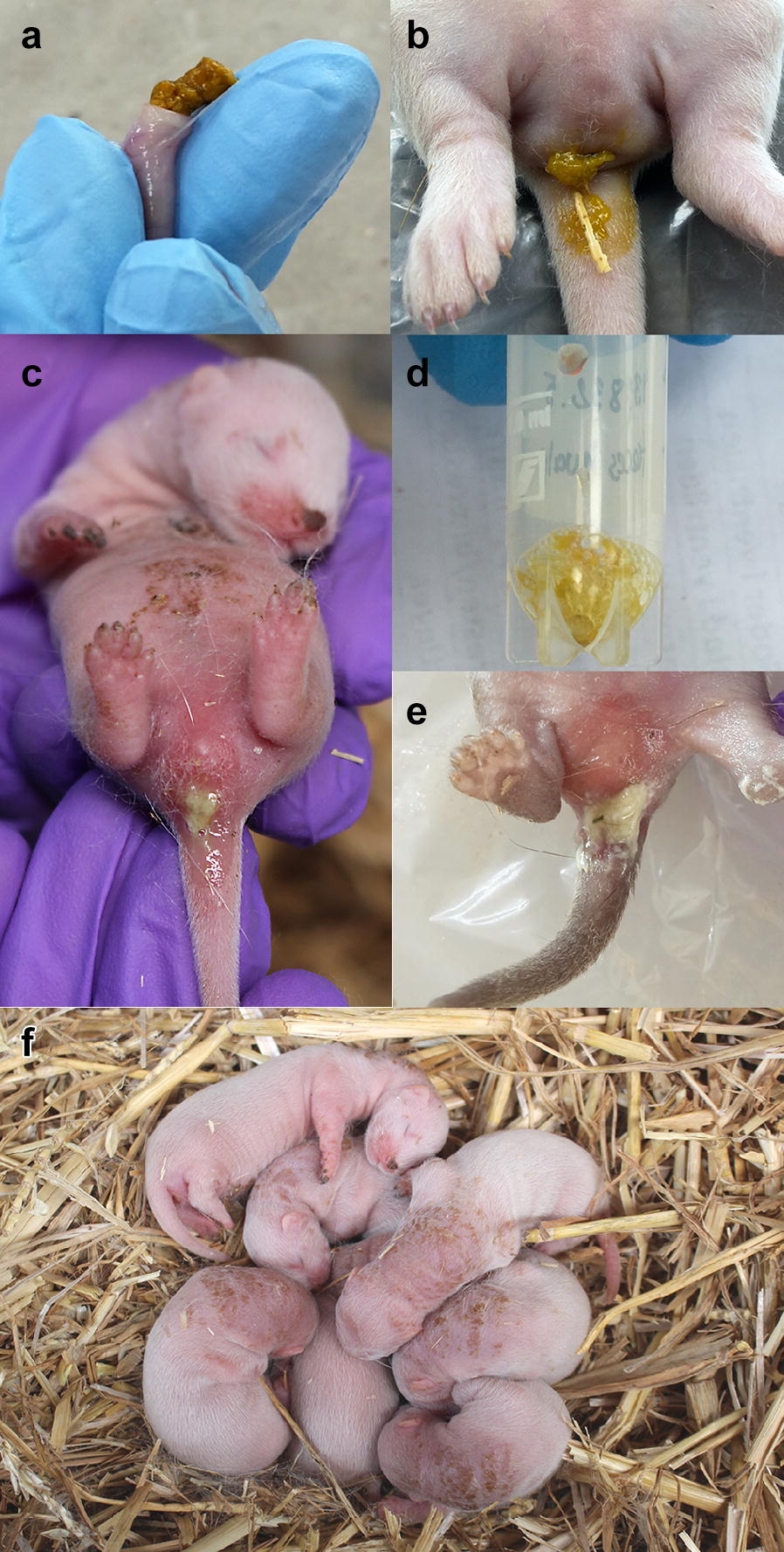
Scoring of rectal contents/feces in mink kits. The numbers refer to the consistency of the feces and the letter to the color. **a** Score 1; Firm to normal soft, log-shaped and moist with smooth surface. **b** Score 2; Soft without shape, very moist, cow-pat like consistency. **c** Score 3; Runny, loose, no defined shape with some texture. Also notice external signs: a sticky exudation on the skin, red swollen anus and black claws. **d** Score 4; Liquid, not containing any particular matter, no texture and may be foamy. **e** Score a; Undigested, white or beige color. **f** A mink litter affected with PWD and cutaneous exudation located to the neck, legs and paws

During the necropsy of the mink kits, signs and assessment of PWD were recorded. The PWD status (PWD±) of the mink litter was based on individual assessments of the intestinal contents in the rectum of the mink kits, combined with the external signs. The definition of PWD status of the mink kit is shown in Fig. 1 and Table 1. Litter PWD status for all litters included in this study was based on concordant kit PWD status of the kits within each litter. The intestinal contents were scored in terms of consistency (score 1–4) and color (score a) as follows; (1) firm to normal soft, log-shaped, moist with a smooth surface, (2) soft without shape, very moist with cow-pat consistency, (3) runny, loose intestinal contents with no defined shape but with some texture, (4) liquid, not containing any particular matter, no texture, may be foamy, (a) if white or beige color as a sign of undigested milk. Kits with score “3”, “4” and/or “a”, were judged to have diarrhea without regard to the external signs (swollen red anus, dirty perineal region or cutaneous exudation on head, legs, trunk, tail or paws). Kits with score “1” or “2” and no external signs were considered as not to having diarrhea. Kits with score “2” and any of the external signs were judged as diarrheic. If no intestinal contents in rectum were present and feces from post mortem defecation was not available, the PWD status was based on the external signs and scores of the litter mates.

**Table 1:** Definition of disease status

PWD+ mink kit		PWD− mink kit	
Feces score^a^	If score 3 OR If score 4 OR If score a	Feces score^a^	If score 1 OR If score 2
			AND
		External assessment	No red or swollen anus AND No dirty perineal region AND No cutaneous exudation on head, legs, trunk tail or paws
If empty rectum or no feces to evaluate OR score 2 (cow-pat like feces)	If swollen or red anus OR If dirty perineal region OR If cutaneous exudation on head, leg, trunk, tail or paws	If empty rectum or no feces to evaluate	If no red or swollen anus AND No dirty perineal region AND No cutaneous exudation on the head, legs, trunk, tail, or paws
			AND litter mates have score 1

^a^Evaluated in rectum or from post mortem “defecation”. A description of the scoring system can be found in Fig. 1

### Assessment of mastitis

#### Bacteriological assessments

The skin covering the mammary glands was cut and peeled off. From each dam, the mammary gland revealing most intensive signs of inflammation (redness, induration and swelling) was opened by a transverse cut with a sterile scalpel and swabbed for bacterial isolation (Transport Swabs, VWR, Radnor, PA, USA). If no signs of inflammation were present, a random gland was sampled. Swabs from the mammary gland tissue and the intestinal contents of the mink kits were transported to Section of Veterinary Clinical Microbiology, University of Copenhagen, where they were inoculated on blood agar [blood agar base (Oxoid, Thermo Scientific, Waltham, MA, US)] enriched with 5% sterile bovine blood. The agar plates were incubated aerobically at 37 °C for 1–2 days. Bacterial colonies were sub-cultured and identified with Matrix-Assisted Laser Desorption Ionization Time-Of-Flight mass spectrometry (MALDI-TOF–MS) using a VITEK MS MALDI-TOF (BioMérieux, Marcy-l’Etoile, France) as described elsewhere [23]. From the opened rectum of one random kit from each litter, a swab was taken from the intestinal contents for bacterial isolation.

### Histopathological assessments

A sample from the previously swabbed gland was fixed in 10% neutral buffered formalin in a CellStor Pot (CellPath, Newtown, Powys, UK). Formalin fixed mammary tissue specimens were cut and embedded in paraffin before sections of 4–5 µm were made and stained with hematoxylin and eosin. During the examination of sections, the following manifestations were graded blindly by JMB using light microscopy: (1) edema (±), (2) number of interstitial foci of calcification in the connective tissue (> 2/≤ 2 per section), (3) number of corpora amylaceae (> 4/≤ 4 per section), (4) presence of excess fibrous tissue (±). Where present, infiltration of mononuclear leucocytes (MNL) and polymorphonuclear leucocytes (PNL) were categorized as focal, multifocal or dispersed confluent in both extra-lobular and intra-lobular regions. A common assessment of intra-lobular and extra-lobular regions regarding infiltration of MNL was defined as ‘few’ (absent or focal) or ‘more’ (multi-focal or dispersed confluent), and for PNL, it was judged as absent or present (focally, multifocally or dispersed confluent).

### Assessment of other risk factors for PWD

#### Herd

The prevalence of mink litters with PWD in the nursing period was used to categorize the farms into high (> 10%) versus low (≤ 10%) prevalence groups.

#### Age of the dam

Data regarding age of the dam was collected on the day of sampling.

#### Litter size

Data regarding the number of live mink kits observed after birth and at the day of sampling were collected.

#### Body mass index (BMI) of the dam

Length from nose to anus and weight of the dams were measured. Body mass index (BMI) was calculated by the formula: BMI = weight(kg)/length(m)^3^ as previously described [24, 25].

#### Total weight of mammary gland tissue

From each dam all the mammary gland tissue was isolated and weighed.

#### Number of mammary glands

The number of active mammary glands was determined by evaluating at the mammary papillae and the volume of the glands.

#### Other concomitant diseases

If present, macroscopic lesions in the abdomen and thorax and external fecal soiling were recorded.

### Statistical analyses

The analytical strategy included univariate analyses of each variable, univariable analysis of association and multivariable analysis of association between PWD and explanatory variables. Descriptive statistics were done as univariate analysis of the response variable and all explanatory variables individually noting if recording errors were present by investigating extreme values, estimation of means and standard deviations and evaluation of the distribution for quantitative variables, and frequency tabulations for categorical variables using proc univariate in SAS v. 9.4 (SAS Institute, Cary, NC, USA); these data are not shown. The analytical unit was the mink litter. The outcome variable was disease status of the litter (PWD+ or PWD−). Due to the limited number of dams and their litters in the study (n = 70), the unbalanced data and to avoid issues of modeling non-linear relationships and to avoid excessive numbers of categories we dichotomized all explanatory variables. The explanatory variable “age” was categorized as “1 year” and “> 1 year”, because it is known from previous studies that there was a higher frequency of PWD among the young than among the old dams [15], and because the farmers at the end of the season the previous year usually keep the best 1 year old dams for next years breeding stock, i.e. selective survival. The number of kits observed after birth was categorized as “≤ 5 kits” and “> 5 kits”, because the national average of kits per litter is around 5. The variable BMI was categorized as “≤ 17.7” and “> 17.7” using the mean value as cut off because there was no other biological good choice. The weight of the mammary glands and the number of active mammary glands were divided by the number of living kits at the day of examination to compensate for the influence of the number of kits had on these variables. The number of mammary glands per kit was categorized as “≤ 1.5” and “> 1.5” because this is around the average number of glands per kit according to the general number of 8 glands per dam and the average number of 5 kits in a litter in the population. The weight of the mammary gland per kit was dichotomized with a cutoff at the average of the two group means (PWD+ and PWD−, respectively), because there was no other biological good choice. Thus the categories were ≤ 12.19 g versus > 12.19 g gland per kit. Bacterial growth of the mammary gland was categorized as positive or negative. The variables concerning the histopathologic evaluation were categorized as described under “Histopathological assessments”.

The statistical analyses for associations between the binary outcome of mink litter disease status (PWD+ and PWD−) and explanatory variables were performed using mixed logistic regression model. Due to the study design, farm-level PWD status (low vs. high) as a fixed effect and farm id as a random intercept were forced into all models. Initially, all other explanatory variables were examined, while only controlling for farm effects and farm-level PWD-status, for their association with litter PWD-status. Subsequently, a final multivariable mixed logistic regression model was fitted. The models were fitted using proc glimmix (SAS) with specification of the logit link function and the binominal distribution. Thus, all analyses were conducted as mixed model logistic regression according to the following generalized linear mixed model equation:

$${\text{Logit}}\left( {{\text{p}}_{\text{ij}} } \right)\, = \,\upbeta0\, + \,\upbeta 1 {\text{ X1}}_{\text{ij}} \, + \, \cdots \, + \,\upbeta{\text{k Xk}}_{\text{ij}} \, + \,\upmu{\text{farm}}_{\text{j}} ,$$

where µfarm is the random effect (variance component) of the individual farm containing (dam + litter)_ij_ and is assumed to be normally distributed with a constant variance (µfarm ~ N(0, σ^2^farm)). The X_ij_’s are the predictor values for the i’th dam (and litter), in the j’th farm, and the relationship between the probability p_ij_ and the binary outcome Y_i_ is unchanged: p(Y_i_ = 1) = pi [26]. Variable selection was done in a backward manual elimination process using the − 2Log Pseudo-Likelihood estimates for comparison of the nested models. The overall model-fit was based on the general-Chi-square/DF being close to 1 [27]. The contribution of each explanatory variable to the model was evaluated as marginal sums of squares and the associated F-test of fixed effects using the Type I error cutoff at α = 0.05. At each modeling step the variable with the highest P-value above α = 0.05 was eliminated. The default estimation method of the generalized linear mixed model was the Residual Log Pseudo Likelihood. Other estimation methods were also assessed (MSPL, RMPL, MMPL and Laplace), but they did not improve the fit of the model assessed on the general-Chi-square/DF. Therefore, the default estimation method was chosen. The final model was assessed using Pearson standardized residual plots. The impact of identified observations with extreme residual value was assessed by comparing the models with and without this observation. All multivariable models are presented with odds ratio estimates and associated P-values and 95% confidence intervals (2 decimals) for the explanatory variables. A test for collinearity between the explanatory variables in the final model was assessed by estimating the Phi coefficient, similar to the Pearson’s correlation coefficient. After identification of the final set of significant variables addition of all two-way interaction terms to the model, one at a time, were evaluated. Presence of confounding in this multivariable model was assessed by comparing the adjusted OR estimates in this model to the crude OR estimated in models with only one exposure variable. A change in the OR estimate > 20% was considered and indication of confounding. Thus, all the variables that were stratified for were considered confounders.

## Results

### PWD status

In total 70 dams with litters were sampled. Based on the definitions of litter PWD status, the PWD+ group consisted of 29 litters and the PWD− group consisted of 41 litters.

### Mastitis

#### Bacteriological findings

In total, 12.9% (9/70) of the bacterial swabs obtained from mammary tissue were cultured positive, and 87.1% (61/70) were negative. In total, 20.7% (6/29) of the swabs were cultured positive in the PWD+ group compared to 7.3% (3/41) in the PWD− group. As presented in Table 2 various bacterial strains were isolated, and strains isolated from the mammary glands did not match strains isolated from the intestines of the kits.

**Table 2:** Bacteria isolated from mink mammary tissue and intestines of their respective kits

Farm ID	Female ID	Isolate from mammary tissue	Diarrhea	Isolates from kits intestines^a^
2	1	*Staphylococcus intermedius* group	−	Not conducted
4	2	*Staphylococcus intermedius* group	−	Unspecific mixture
4	3	*Staphylococcus lentus*	+	Non-hem. *Escherichia coli*
4	4	*Micrococcus luteus*	+	Non-hem. *Escherichia coli*
12	5	*Staphylococcus lentus*	+	Non-hem. *Escherichia coli*
13	6	*Staphylococcus aureus*	+	Overgrowth of *Proteus* spp.
19	7	*Staphylococcus intermedius* group	−	Not conducted
25	8	*Enterococcus hirae*	+	Hemolytic *Escherichia coli*
28	9	*Staphylococcus intermedius* group	+	*Enterococcus hirae*

Types of bacteria isolated by aerobic cultivation from mink dam mammary tissue (n = 9) and from intestines of their respective mink kits

^a^The most dominant species

### Histological findings

**Fig. 2:**
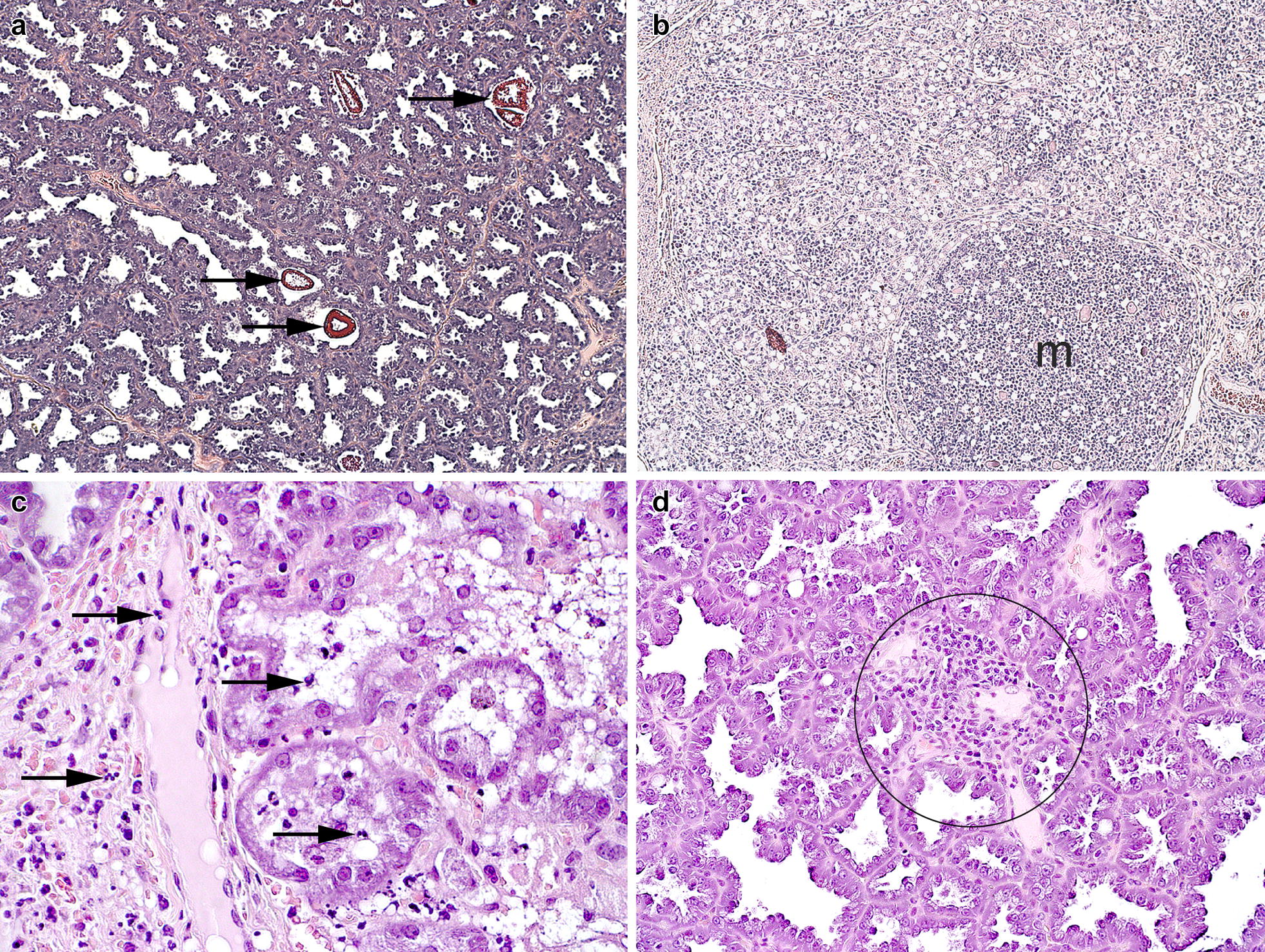
Examples of histopathological findings in mammary gland tissue from mink dams. **a** Non-infiltrated gland tissue with several corpora amylacea (milkstones) (arrows). **b** Disseminated infiltration of polymorphonuclear leucocytes with microabscess formation (m). **c** Infiltration with polymorphonuclear leucocytes (arrows) in the connective tissue and lumen of acini with disruption of the normal architecture. **d** Focal intralobular infiltration of mononuclear leucocytes (circle)

Results from the histopathologic evaluation of mammary tissue samples are shown in Table 3 and examples of findings are presented in Fig. 2. In total, 44.8% (13/29) of the samples had “more” infiltration of MNL in the PWD+ group compared to 39.0% (16/41) in the PWD− group, and 20.7% (6/29) of the samples had infiltration with PNL in the PWD+ group, compared to 17.1% (7/41) in the PWD− group. Edema was present in 37.9% (11/29) of the samples in the PWD+ group compared to 36.6% (15/41) in the PWD− group, whereas excess fibrous tissue was rather rare and present in 6.9% (2/29) and 4.9% (2/41) of the samples in the PWD+ group and PWD− group, respectively. Other lesions included corpora amylacea, which were present in high frequency (> 4/slide) in 10.3% (3/29) of the samples in the PWD+ group compared to 17.1% (7/41) in the PWD− group. Mammary tissue calcification was not as common as corpora amylacea and present in high frequency (> 2/slide) in one sample from each group (3.4% and 2.4% in the PWD+ and PWD− group, respectively).

**Table 3:** Associations between litter PWD status and individual dam- and litter-level risk factors after controlling for farm and design effects

Exposure variable	N	Fixed effect of the exposure variable in the model					Fixed effect of the extraneous design variable		
		PWD+	PWD−	OR	CI_95%_ of OR	P	High vs. low farm PWD prevalence^a^		
							OR	CI_95%_ of OR	P
Only farm PWD status in the model	70			–	–	–	8.61	2.29; 32.30	0.002
Female characteristics									
Age of dam (1-year/> 1-year)	70^b^	25/4	20/21	8.54	1.97; 36.98	0.005	10.10	2.55; 40.05	0.002
Number of kits observed after birth (> 5/≤ 5)	70	15/14	2/39	25.00	3.91; 159.68	0.001	8.88	2.13; 37.00	0.004
Body mass index^b^ (≤ 17.7/> 17.7)	70	14/15	24/17	0.62	0.19; 2.02	0.42	8.80	2.31; 33.56	0.002
Number of active glands per mink kit (≤ 1.5/> 1.5)	70	15/14	10/31	4.92	1.29; 18.79	0.02	11.14	2.69; 46.10	0.001
Weight of mammary gland tissue per kit (g) (> 12.19/≤ 12.19)	68^c^	8/20	18/22	2.02	0.58; 7.00	0.26	9.61	2.46; 37.5	0.002
Bacteriology of mammary gland									
Bacterial growth of mammary gland (±)	70	6/23	3/38	9.08	1.25; 65.71	0.03	13.19	3.03; 57.38	0.001
Histology of mammary gland									
Infiltration of MNL (more/few)	70	13/16	16/25	0.80	0.24; 2.68	0.72	9.16	2.32; 36.18	0.002
Infiltration of PNL (present/absent)	70	6/23	7/34	1.82	0.37; 9.01	0.45	9.30	2.36; 36.61	0.002
Mammary tissue oedema (±)	70	11/18	15/26	0.72	0.21; 2.45	0.60	9.22	2.35; 36.18	0.002
Mammary tissue calcification (> 2/≤ 2 per slide)	70	1/28	1/40	2.36	0.07; 76.69	0.62	8.91	2.29; 34.63	0.002
Mammary corpora amylacea (> 4/≤ 4 per slide)	70	3/26	7/34	0.72	0.12; 4.36	0.72	8.56	2.24; 32.68	0.002
Excess fibrous connective tissue (±)	70	2/27	2/39	0.47	0.05; 4.94	0.52	9.76	2.42; 39.45	0.002

All models included Farm ID as random effect variable. All models included two fixed effects explanatory variables of which the extraneous design variable “Farm PWD status” with high farm prevalence vs. low farm prevalence of litters with PWD was forced into each model and one exposure variable of interest, e.g. “Age of dam”. Each line represents one model

*BMI* body mass index, *MNL* mononuclear leucocytes, *PNL* polymorphonuclear leucocytes

^a^ >/<10% prevalence affected litters

^b^The 70 litters in the analysis are dichotomized according the the exposure level and disease status. Thus, as an example, the model with “Age of dam” had 25, 4, 20 and 21 litters in the categories and sums up to 70

^c^Information was missing for two dams

### Other risk factors for PWD

#### Age of the dam

Out of 70 dams included in the study 45 (64.3%) were young (1-year) and 25 dams were older. The proportion on young dams was 86.2% (25/29) in the PWD group whereas young dams accounted for 48.8% (20/41) in the PWD− group.

#### Litter size

The median litter size defined as the number of kits observed after birth was 5.5 (range 2–10, SD = 2.4) in the PWD+ group and 3.2 (range 1–7, SD = 1.2) in the PWD− group. When dichotomized, large litter size (> 5 kits) were present in 51.7% (15/29) of the litters in the PWD+ group whereas the proportion was only 4.9% (2/41) in the PWD− group.

#### Body mass index (BMI) of the dam

The mean BMI was 18.0 (SD = 2.4) and 17.5 (SD = 2.0) in the PWD+ and PWD− group, respectively. When dichotomizing at the grand mean BMI = 17.7, BMI above 17.7 was present in 51.7% (15/29) of the dams in the PWD+ group compared to 41.5% (17/41) in the PWD− group.

#### Weight of mammary gland tissues per kit

The mean weight of the mammary gland tissue per kit was 11.5 g (SD = 4.5) and 12.7 g (SD = 4.2) in the PWD+ and PWD− dams, respectively. When dichotomized, weight of mammary tissue per kit > 12.19 g was present in 28.6% (8/28) in dams in the PWD+ group compared to 45% (18/40) in the PWD− group.

#### Number of mammary glands

The median number of active mammary glands per kit was 1.5 (SD = 0.5) and 1.8 (SD = 0.43) in the PWD+ and PWD− group, respectively. Hence, the dams provided ≤ 1.5 mammary gland per kit in 51.7% of the PWD+ litters and 24.4% of the PWD− litters.

#### Other concomitant diseases

The necropsies revealed only few pathological findings; 3 dams with perineal soiling as a sign of diarrhea in the PWD+ group, one dam with splenomegaly (PWD+ group), one dam from each group with uterus contents (hydrometra and retained placenta) and one dam with ventricle ulcer (PWD− group).

### Associations between PWD and the explanatory variables

The multivariable models for the associations between PWD and dam characteristics, histological changes and bacteriology are presented in Table 3. There was a statistically significant association between the extraneous variable “farm PWD status” and the litter PWD status in all 13 models in Table 3. Only in four of the models there was a statistically significant association between the litter PWD status and the exposure variable of primary interest. Hence, the odds of a litter having PWD were significantly higher in litters from young females, in litters with high litter size, in litters with fewer active mammary glands per kit and in litters from females with positive bacterial growth from the mammary gland (Table 3).

The final multivariable logistic regression model is presented in Table 4 with OR, 95% confidence intervals and P-values. The odds of having PWD in the mink litter were significantly greater in the following: litters from a young dam, large litters, litters with a dam with ≤ 1.5 active mammary glands per kit, and if the litters were from a farm with a high prevalence of PWD (Table 4). Farm-id was included as random effect. Thus, the variables concerning bacterial infection or histopathologic signs of mastitis or other mammary lesions were not included in the final model as explanatory variables for PWD in the litter. None of the two-way interaction terms between the four fixed effects variables in the model contributed statistically significantly to the model fit. Confounding was present among all the four fixed effects variables in the model, assessed as a > 20% change in the OR estimated from the crude odds ratio to the final model adjusted ORs. Regression diagnostics showed that the model fitted the data as the Generalized Chi Square = 1.11 was considered close to one, and thus there was no residual overdispersion. Pearson standardized residual plots showed a linear prediction close to “0” and a close to normal distribution except for a single residual outlier. Exclusion of this outlier observation neither changed the model nor the model fit, and therefore the observation was kept in the dataset.

**Table 4:** Multivariable mixed effects logistic regression model for associations between litters with and without PWD and four exposure variables as fixed effects and farm-id as random effect

Variable	Odds ratios		P-value for marginal sums squares F-test
	Estimate	95% confidence limits	
Age of dam (1-year vs. > 1 year)	13.3	2.0; 90.2	0.009
Number of kits observed after birth (> 5 vs. ≤ 5)	16.5	2.2; 123.7	0.008
Number of active mammary glands per kit (≤ 1.5 vs. > 1.5)	6.5	1.2; 36.0	0.034
Farm PWD status (high vs. low frequency)	16.8	2.9; 97.6	0.002

The intra class correlation coefficient ICC = 0.245. The model fits the data as the generalized Chi-square/DF = 1.11 and hence being reasonably close to 1

## Discussion

We found that PWD in the mink litter was associated with the age of the dam, litter size, the number of active mammary glands per kit and the farms PWD status. Odds of PWD were significantly higher in litters from 1-year old dams compared to older dams. This is consistent with previous studies showing that dams of first year had a higher risk of PWD in the litter compared to older dams [15, 28]. A speculated reason for this might be a lower quality of nursing skills in young dams or a lesser transfer of maternal immunity to the kits. However, mink are like other production animals selected for high performance such as reproduction, growth and pelt quality; hence, older dams are expected to be more robust animals to remain in the herd which may also explain this effect of dam age. High litter size also increased the odds of PWD in the mink litter, which is in line with previous studies [15, 28]. This may have caused a more competitive pressure among the kits in the litter and may have resulted in different kinds of stress. However, in our study we only used the dam and 2–3 kits from each litter. The remaining kits in the litter were after euthanization of the dam reallocated to other dams in the farm. To reduce this need for fostering care of remaining mink kits, mink farmers probably tended to present smaller litters to the investigation team at the time of sampling. We do not know the sampling fractions of large (< 5) and small (≤ 5) litters in the farms, but believe the sampling fractions of large litters were lower than of small litters. This is also reflected in the mean litter sizes of PWD+ and PWD− litters, i.e. 5.5 and 3.2 kits per litter, respectively. If this is true, the ratio of sampling fractions [26] for large and small litters among PWD+ and among PWD− litters are equally affected. Therefore, the estimated odds ratio for association between litter size and PWD in the final model is believed to be close to the true population odds ratio, and thus not selection biased. Odds of PWD were higher among litters with fewer active mammary glands per kit. This finding suggests that mink kits in litters with restricted access to milk supply have increased risk of PWD. This may be due to a decreased transfer of humoral immunity (IgG) by the milk, which recently in mink has been shown to be litter specific and to some extend correlated to the IgG levels in the dam [17]. The statistically significant contribution to the multivariable model of ‘farm PWD status’ is very likely a proxy for the presence of other important risk factors on the farms than the ones included in our study, e.g. management regarding feeding, hygiene and biosecurity.

We examined the association between PWD and the presence of bacterial infection and microscopic lesions in the mammary gland of the dam. Bacteria were grown from the mammary glands of 12.9% of the dams, and since the majority of the samples were culture negative, it indicates that our sampling technique was adequate to avoid contamination. Isolates from the *S. intermedius* group were most commonly identified, and many of the cultures were monocultures which most likely reflect a true ascending infection of the mammary gland. Isolates belonging to the *S. intermedius* group from mink is most likely represented by *S. delphini* group A, which has been isolated from different body locations and is a normal inhabitant in mustelids [12]. We did not identify *E. coli* from mammary glands which, however, is a quite common isolate from mink mammary tissue from dead or euthanized dams sent for routine laboratory diagnostics (National Veterinary Institute, Denmark, unpublished). One reason for this difference may be that the population of dams in the present study may represent a broader spectrum of less severe mammary infection. There was not a significant association in the multivariable model between PWD and growth of bacteria in the mammary gland of the dam. Neither could we demonstrate dominance of the same bacterial species in the gut of the respective mink kits, which also counts against an association between PWD and mammary infection. Bacterial isolation from the mammary gland does not necessarily implicate inflammation as isolates may be recovered from dormancy or are newly established. It is likely that some bacterial strains, e.g. *S. intermedius* group or *S. aureus* are more pathogenic than others and result in more inflammation which could be of interest in future studies. However, PWD was not associated with inflammation defined as infiltration of PNL or MNL, and other lesions of the mammary gland, which is in line with another study where no association between PWD and mastitis was found [22]. The implications of finding no relation between PWD and mastitis suggest that PWD occurs independently of mastitis. However, a general weakening of the immune system of the mink dams might result in a higher prevalence of both PWD and mastitis on certain farms. A shortcoming of this study is the one-time investigation without the opportunity to follow-up, during a period covering an incubation period. Furthermore, we did not examine all mammary glands from the dams, but selected those suspected of being inflamed. Material for this study was collected from commercial mink farms and was dependent on spontaneous outbreaks of PWD. Sample size calculations were impossible to make due to lack of information on associations to risk factors examined, and this might have resulted in a lack of statistical power. However, we consider not having selection bias due to convenience sampling of farms. The farms included in this study were selected from different Danish geographical regions, supplied by different feed producers and with various veterinarian advisors and thus believed to be representative of commercial Danish AMDV-free mink farms. The high and low prevalence farms are, therefore, believed to be representative of the farm populations with high and low prevalence of litters with PWD. Our study includes the most important risk factors, based on literature and our biological knowledge. This paper has focused on dam-related characteristics associated with PWD, which are relevant because the mink kits at risk of PWD are in the suckling state of life and totally dependent on the dam. However, more studies are needed to elucidate the etiology of this syndrome. Microbiological analyses of material collected from the examined mink kits will be reported elsewhere.

## Conclusions

Pre-weaning diarrhea was associated with farm PWD status, age of the dam, number of kits observed after birth and the number of active mammary glands per kit. We did not find an association between PWD and mammary gland bacterial infection or histological lesions as indicators of mastitis.

## Abbreviations

BMI: body mass index
MNL: mononuclear leucocytes
OR: odds ratio
PNL: polymorphonuclear leucocytes
PWD: pre-weaning diarrhea
SIG: *Staphylococcus intermedius* group

## References

[1] Clausen TN, Dietz HH (2004). Wet kits in mink, a review. Scientifur.

[2] Henriksen P. “Wet mink kits”—acute enteritis in pre-weaning mink. In: Proc. IV Int. Congr. Fur Anim. Prod. Alberta: IFASA; 1988. p. 208–12.

[3] Englund L, Chriél M, Dietz HH, Hedlund K-O (2002). Astrovirus epidemiologically linked to pre-weaning diarrhoea in mink. Vet Microbiol.

[4] Svansson V. Study of a number of virus-induced infections in mink [in Danish]. Doctoral Thesis. University of Copenhagen; 1991.

[5] Hansen S. Application of qPCR, pathologic examination and electron microscopy for diagnostic investigation of astro-, corona- and rotavirus in farmed mink (*Neovison vison*). Master Thesis. University of Copenhagen; 2014.

[6] Guo M, Evermann JF, Saif LJ (2001). Detection and molecular characterization of cultivable caliciviruses from clinically normal mink and enteric caliciviruses associated with diarrhea in mink. Arch Virol.

[7] Jørgensen M, Scheutz F, Strandbygaard B (1996). *Escherichia coli* and virus isolated from “sticky kits”. Acta Vet Scand.

[8] Danieu P, Anderson B, Maes R, Bolin C, Kiupel M. “Sticky kits” syndrome in mink (*Mustela vison* L.): A secretory diarrhea associated with *Staphylococcus intermedius* colonization. In: Proceedings of the Institute for Zoo and Wildlife Research, no 6; 2005. p. 122–3.

[9] Sledge DG, Danieu PK, Bolin CA, Bolin SR, Lim A, Anderson BC (2010). Outbreak of neonatal diarrhea in farmed mink kits (*Mustella vison*) associated with enterotoxigenic *Staphylococcus delphini*. Vet Pathol.

[10] Vulfson L, Pedersen K, Chriél M, Frydendahl K, Holmen Andersen T, Madsen M (2001). Serogroups and antimicrobial susceptibility among *Escherichia coli* isolated from farmed mink (*Mustela vison Schreiber*) in Denmark. Vet Microbiol.

[11] Sasaki T, Kikuchi K, Tanaka Y, Takahashi N, Kamata S, Hiramatsu K (2007). Reclassification of phenotypically identified *Staphylococcus intermedius* strains. J Clin Microbiol.

[12] Guardabassi L, Schmidt KR, Petersen TS, Espinosa-Gongora C, Moodley A, Agersø Y (2012). Mustelidae are natural hosts of *Staphylococcus delphini* group A. Vet Microbiol.

[13] Vulfson L, Pedersen K, Chriel M, Andersen TH, Dietz HH (2003). Assessment of the aerobic faecal microflora in mink (*Mustela vison* Schreiber) with emphasis on *Escherichia coli* and *Staphylococcus intermedius*. Vet Microbiol.

[14] Møller SH, Chriél M (2000). Health effects of the feeding strategies in the pre-mating and gestation periods of mink. Scientifur.

[15] Chriél M (1997). Let the mink females decide them selves. Dansk Pelsdyravl.

[16] Birch JM, Agger JF, Dahlin C, Jensen VF, Hammer AS, Struve T (2017). Risk factors associated with diarrhea in Danish commercial mink (*Neovison vison*) during the pre-weaning period. Acta Vet Scand.

[17] Mathiesen R, Chriél M, Struve T, Heegaard PMH (2018). Quantitative immunoassay for mink immunoglobulin in serum and milk. Acta Vet Scand.

[18] Uttenthal Å, Henriksen P, Østergård J, Clausen T, Costello F. Measurement of immunoglobulins in mink [in Danish]. Annual Report, Kopenhagen Res. Holstebro: Kopenhagen Fur; 1998. p. 119–24.

[19] Hunter DB. Mink-biology, health and disease. In: Lemieux N, editor. Guelp: Graphic and Print Services, University of Guelph; 1996.

[20] Trautwein GW, Helmboldt CF (1966). Mastitis in mink due to *Staphylococcus aureus* and *Escherichia coli*. J Am Vet Med Assoc.

[21] Hammer AS, Sørensen CM, Jensen TH (2008). Mastitis in mink. Fur Anim Res.

[22] Clausen TN, Dietz HH (2000). Mastitis in the lactating mink female (*Mustela vison* S.) and the development of “greasy kits”. Acta Vet Scand.

[23] Fang H, Ohlsson AK, Ullberg M, Özenci V (2012). Evaluation of species-specific PCR, Bruker MS, VITEK MS and the VITEK 2 system for the identification of clinical *Enterococcus* isolates. Eur J Clin Microbiol Infect Dis.

[24] Mustonen AM, Paakkonen T, Ryokkynen A, Asikainen J, Nieminen P, Pyykonen T (2005). Adaptations to fasting in the American mink (*Mustela vison*): carbohydrate and lipid metabolism. Comp Biochem Physiol Part A Mol Integr Physiol.

[25] Nieminen P, Hyvärinen H, Käkelä R, Asikainen J (2000). Plasma leptin and thyroxine of mink (*Mustela vison*) vary with gender, diet and subchronic exposure to PCBs. Comp Biochem Physiol Part A Mol Integr Physiol.

[26] Dohoo I, Martin W, Stryhn H (2010). Veterinary epidemiologic research.

[27] Schabenberger O. Introducing the GLIMMIX procedure for generalized linear mixed models. SUGI 30 Proceedings, Philadelphia, Pennsylvania, April 10–13 2005. Paper 196-30. 20 pages.

[28] Olesen CR, Clausen TN. Sticky kits results from the Investigation Farm West 1989. Annual Report. Kopenhagen Res. Holstebro: Kopenhagen Fur; 1989. p. 154–64 **(in Danish)**.

